# Analytical description of nanowires III: regular cross sections for wurtzite structures

**DOI:** 10.1107/S2052520622004954

**Published:** 2022-07-15

**Authors:** Dirk König, Sean C. Smith

**Affiliations:** a Integrated Materials Design Lab (IMDL), Research School of Physics and Engineering, The Australian National University, ACT 2601, Australia; b Institute of Semiconductor Electronics (IHT), RWTH Aachen University, 52074 Aachen, Germany; c Integrated Materials Design Centre (IMDC), University of New South Wales, NSW 2052, Australia; d Department of Applied Mathematics, Research School of Physics and Engineering, The Australian National University, ACT 2601, Australia; Polish Academy of Sciences, Poland

**Keywords:** nanowires, wurtzite, cross section, analytic description

## Abstract

Setting out from König & Smith [ *Acta Cryst.* (2019), B**75**, 788–802; *Acta Cryst.* (2021), B**77**, 861], we present an analytic description of nominal wurtzite-struc­ture nanowire (NWire) cross sections, focusing on the underlying geo­metric–crystallographic description and on the associated number theory. For NWires with diameter *d*_Wire_[*i*], we predict the number of NWire atoms *N*_Wire_[*i*], the bonds between these *N*_bnd_[*i*] and NWire interface bonds *N*_IF_[*i*] for a slab of unit-cell length, along with basic geometric variables, such as the specific length of interface facets, as well as widths, heights and total area of the cross section. These areas, the ratios of internal bonds per NWire atom, of internal-to-interface bonds and of interface bonds per NWire atom present fundamental tools to interpret *any* spectroscopic data which depend on the diameter and cross section shape of NWires. Our work paves the way for a fourth publication which – in analogy to König & Smith [ *Acta Cryst.* (2022). B**78**, 643–664] – will provide adaptive number series to allow for arbitrary morphing of nominal w-structure NWire cross sections treated herein.

## Abstract 2

Setting out from König & Smith [ *Acta Cryst.* (2019), B**75**, 788–802; *Acta Cryst.* (2021), B**77**, 861], we present analytic descriptions of regular wurtzite-structure nanowire (NWire) cross sections, focusing on the underlying geometric–crystallographic definitions and the associated number theory. We predict the NWire atoms, bonds between these atoms and NWire interface bonds, characteristic lengths and cross section area for a periodic NWire slab. These parameters present fundamental tools to interpret any spectroscopic data which depend on the diameter and cross section shape of NWires.

## 1. Introduction

Recently, we described the cross sections of zincblende (zb) and diamond-structure NWires of regular shape (König & Smith, 2019 ▸, 2021 ▸), extending such analytic crystallographic tools to convex cross sections of arbitrary shape, including irregular multi-core-shell zb-NWires (König & Smith, 2022 ▸). In this work, we introduce a description of regular wurtzite (w-) structure NWire cross sections by an analytic number series in analogy to the above-mentioned publications. While our previous works on regular zb- and diamond-structure NWire cross sections contained a considerable amount of experimental data from the literature to demonstrate the application of such analytic number series, we focus here more on the underlying crystallographic geometry and number theory. The reason for not including experimental data from the literature in our present work is twofold: first, and in contrast to zb-/diamond-structure NWires, there is little published experimental work (if any) which describes *fully regular* w-NWire cross sections in enough detail (*i.e.* with sufficient spatial resolution) to match them with analytic number series. Several literature sources exist for irregular-shaped w-NWire cross sections, consisting of CdS, CdSe (Duan & Lieber, 2000 ▸), GaN (Kuykendall *et al.*, 2004 ▸), GaAs (Zardo *et al.*, 2009 ▸; Harmand *et al.*, 2018 ▸), core-shell GaAs-SiGe (de Matteis *et al.*, 2020 ▸), InAs (Caroff *et al.*, 2009 ▸), InP (Gao *et al.*, 2014 ▸) and Si (Wang *et al.*, 2021 ▸). Second, we received several requests to explain the underlying crystallographic geometry and number theory used to arrive at the equations we pub­lished previously. Therefore, we elaborate on these two topics to explain our method. Such explanations can also be applied to the zb- and diamond-structure NWire cross sections with [111] and 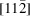 growth vectors we published recently, with some minor modifications in offset areas and lengths.

**Figure 1:**
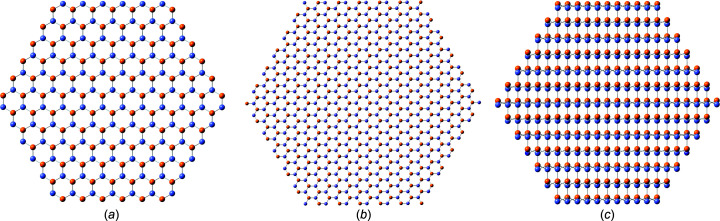
Examples of NWire cross sections with w-structure treated in our work, shown for a binary compound such as GaN: (*a*) [0001] growth vector and six 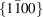 interfaces, (*b*) [0001] growth vector and six {1000} interfaces, and (*c*) 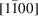 growth vector, {0001} interfaces at the top and bottom, plus four {0021} side interfaces. These cross sections have experimental counterparts and are thus relevant to structural analysis.

We describe three w-NWire cross sections which were shown to exist in experiment as per the references above, namely, w-NWires growing along the [0001] vector with 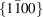 interfaces, w-NWires growing along the [0001] vector with {1000} interfaces and w-NWires growing along the 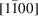 vector with {0001} and {0021} interfaces. Examples of these NWire cross sections are shown in Fig. 1 ▸.

We proceed as follows: Section 2 gives a brief introduction to the wurtzite structure, then providing crystallographic data and the variables of interest with their indices. The number series for generating such variables are presented in Section 3. For each cross section, we introduce an *even* and an *odd* version in analogy to our work on regular cross sections for zb-NWires (König & Smith, 2019 ▸, 2021 ▸), accounting for different sym­metry centres of the NWire to match corresponding cross section images with atomic resolution. We discuss the application of these variables in Section 4 and sum up our findings in Section 5. The Appendices consist of three parts, providing additional input on geometric details for cross sections of w-NWires growing along the [0001] vector (Appendix *A* [App appa]), for the cross section of w-NWires growing along the 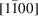 vector (Appendix *B* [App appb]) and for the derivation of all *even* number series of the NWire cross section with a [0001] growth vector and 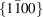 interfaces as an example (Appendix *C* [App appc]).

## 2. General remarks on analytical number series, structural boundary conditions and nomenclature

**Figure 2:**
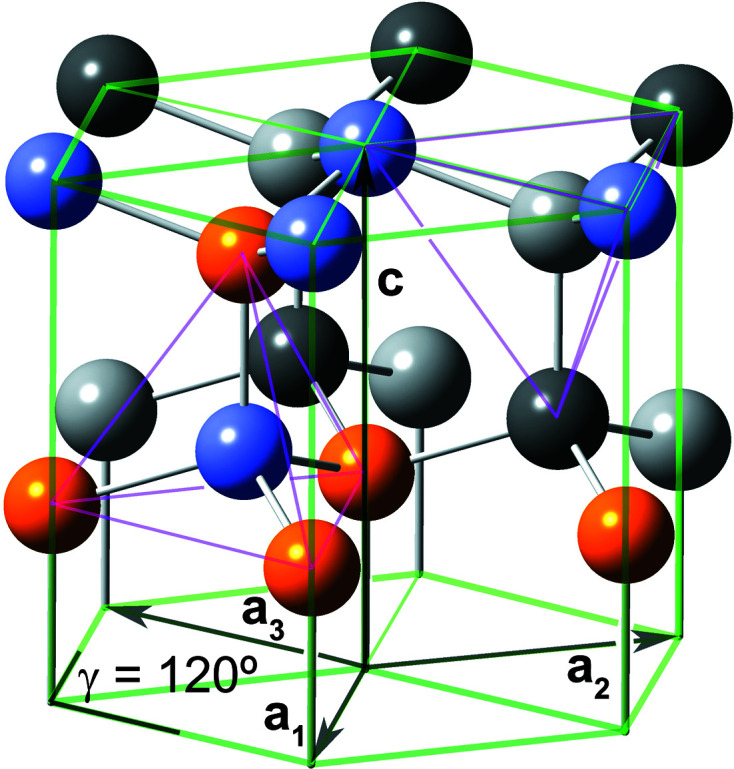
Periodic unit cell (UC) of a wurtzite solid with lattice vectors **a**_1_, **a**_2_ and **a**_3_, *viz.* 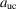 = 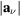 , and **c**, *viz.* 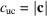 , space group *P*6_3_ *mc* (wurtzite) covered in this work, such as gallium nitride (w-GaN). The orange (Ga) and grey–blue (N) atoms framed in dark green show the primitive UC. The full w-UC is formed by including atoms shaded in light grey (Ga) and dark grey (N), and is outlined in bright green. All atoms at the lateral periodic boundaries, *i.e.* for all 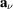 ≠ 0 and 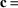 constant, were shown to facilitate UC visualization. The NGa_3_ (left) and GaN_3_ (right) tetrahedra interlinked within a couple of corrugated atomic planes are shown by magenta lines.

Apart from several polar II–VI and III–V semiconductors possessing w-structure symmetry, Si-NWires were observed to expose w-structure symmetry under local stress in de Matteis *et al.* (2020 ▸) or when grown by specific bimetallic catalysts (Wang *et al.*, 2021 ▸). Both material groups share the same crystal symmetry (space group *P*6_3_ *mc*) apart from their primitive basis which is *A*–*B* (Ga–N) or *A*–*A* (Si–Si) (Hammond, 2001 ▸). The w-unit cell (w-UC) is shown in Fig. 2 ▸.

Defect-free crystalline NWires have a one-dimensional periodicity along their growth axis, enabling their cross section to be described by a disk with a thickness 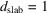 UC in the respective growth direction. For the two cross sections with a [0001] growth vector, this thickness is given by 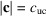 (see Fig. 2 ▸). For the remaining cross section with a 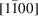 growth vector, we obtain 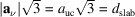 ; see side views of the cross sections in Figs. 7 and 8. The number of atoms per atom column and the number of bonds within and between these naturally depend on 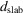 and the growth vector. We listed both parameters along with 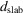 in Table 1 ▸, with a diagrammed version for the bonds to allow for an easy interpretation of the respective cross sections for geometrical analysis and number theory in Section 3.

Next, we introduce the variables we describe analytically per NWire cross section. The first group of variables describes the atoms or bonds (internal, interfacial) constituting the NWire slab. The second group contains all variables which provide spatial information, such as width, height, interface lengths and cross section area (Table 2 ▸).

Finally, we need a clear nomenclature for the respective growth vector and interface orientations to distinguish the above variables. Such indices are given in Table 3 ▸.

Although all NWire cross sections in our work have a hexagonal shape, their direct comparison per NWire size is most appropriately done by calculating their diameter, assuming a circular shape of the cross section *via*


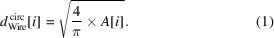

Values of 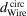 will become relevant in Section 4.

## 3. Analytical number series of nanowire cross sections

As we demonstrated in our previous work concerning zb-NWire cross sections (König & Smith, 2019 ▸, 2021 ▸) with experimental data (Yi *et al.*, 2011 ▸), it is a great advantage to have two different descriptions per cross section, each featuring a distinct symmetry centre. To this end, we introduce an *even* and an *odd* version for each cross section in analogy to our work mentioned above, accounting for different symmetry centres of the NWire to match corresponding cross section images with atomic resolution. Both the *even* and *odd* versions for each cross section are covered in the same section below. For brevity, we keep the description to a minimum and only add information where essential.

### NWires growing along the [0001] direction with a hexagonal cross section and six 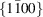 interfaces

3.1.

Additional information on the geometric details of the wurt­zite structure for calculating offsets in characteristic lengths and areas of this cross section is given in Appendix *A* [App appa].

We start with the *even* series of this NWire cross section. A detailed numerical derivation of Equations 2–8 [Disp-formula fd3] [Disp-formula fd4] [Disp-formula fd5] [Disp-formula fd6] [Disp-formula fd7] [Disp-formula fd8] below is given in Appendix *C* [App appc] as an example. 

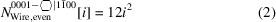




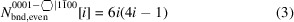




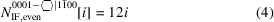




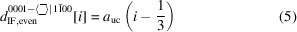




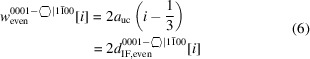




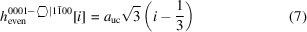




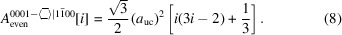

**Figure 3:**
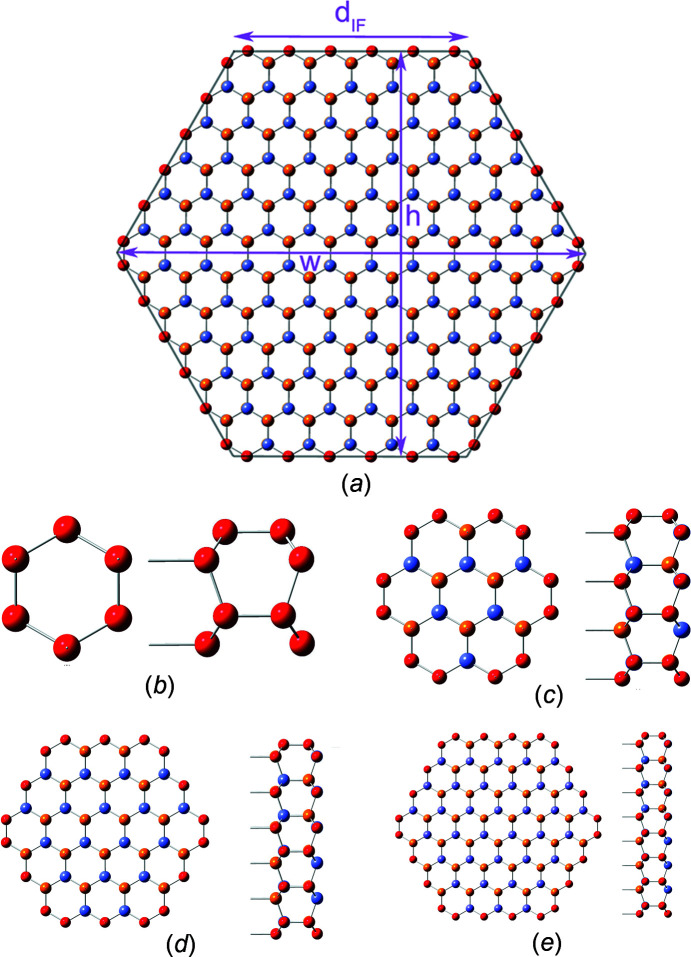
(*a*) Definition of the characteristic lengths for the w-structured NWires growing along the [0001] axis with a hexagonal cross section and six 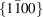 interfaces, which are shown by translucent black lines. The six isoceles triangles located at the six corners of the cross section present the offset area and offsets of interface lengths which are all constant for all cross sections of this type, applying to *even* and *odd* series alike. (*b*)–(*e*) Top and side views of the first four members, *even* series: (*b*) X_12_ (*i* = 1), (*c*) X_48_ (*i* = 2), (*d*) X_108_ (*i* = 3) and (*e*) X_192_ (*i* = 4). The colours of the internal atoms are orange for Ga and grey–blue for N. Red atoms have one interface bond.

The definition of interface boundaries for the calculation of characteristic lengths and the cross section area is shown in Fig. 3 ▸, together with top and side views of the cross sections for the first four members of the *even* number series.

We now list the *odd* series of the cross sections with a [0001] growth vector and 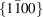 interfaces. 

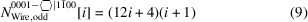




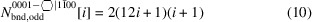




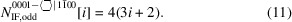

 From Equation 11, we see that 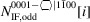 = 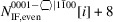 , accounting for the elongated form of the *odd* series cross sections. 

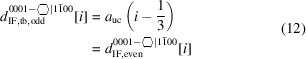




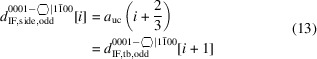




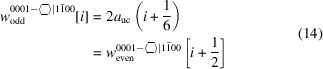




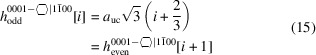




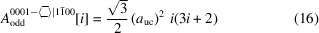

**Figure 4:**
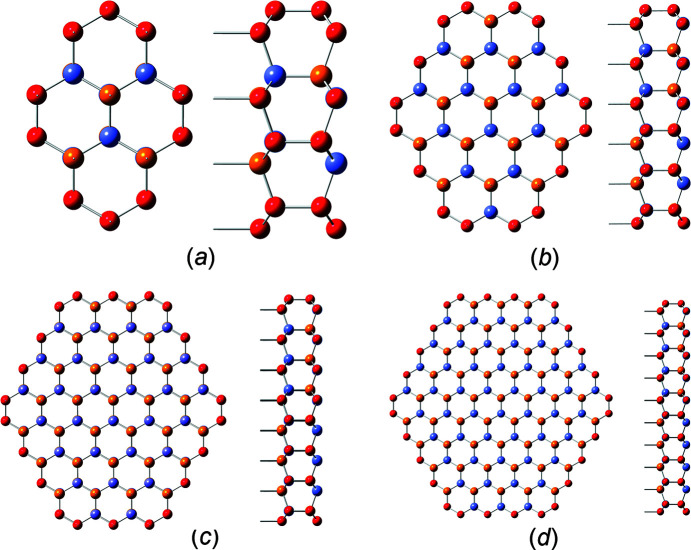
Top and side views of the first four members of w-structured NWires growing along the [0001] axis with a hexagonal cross section and six 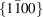 interfaces, *odd* series: (*a*) X_32_ (*i* = 1), (*b*) X_84_ (*i* = 2), (*c*) X_160_ (*i* = 3) and (*d*) X_260_ (*i* = 4). For atom colours, see Fig. 3 ▸.

The first four members of the *odd* number series are shown in Fig. 4 ▸.

### 3.2. NWires growing along the [0001] direction with a hexagonal cross section and six {1000} interfaces

This cross section is more straightforward in that it does not have any offsets in characteristic lengths or cross section area. As before, we start with the *even* series of this cross section. 

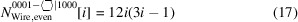




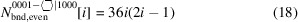




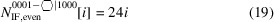




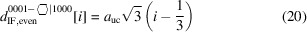




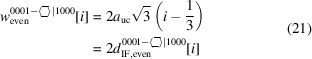




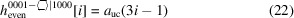




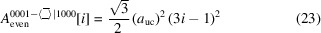

**Figure 5:**
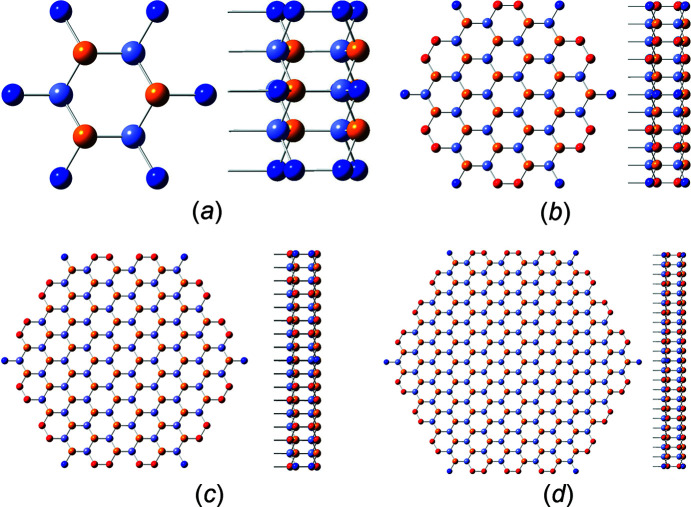
Cross section and side view of w-structured NWires with a [0001] growth axis, hexagonal cross section and {1000}-oriented interfaces, *even* series: (*a*) X_24_ (*i* = 1), (*b*) X_120_ (*i* = 2), (*c*) X_288_ (*i* = 3) and (*d*) X_528_ (*i* = 4). We skipped the assignment of interface lengths, width, and the height of this cross section type as these can be seen in a straightforward manner. Red atoms have one interface bond and blue atoms have two interface bonds.

The first four members of the *even* number series are shown in Fig. 5 ▸.

We now list the *odd* series of the cross sections with a [0001] growth vector and 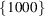 interfaces. 

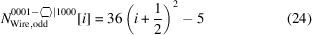




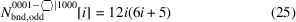




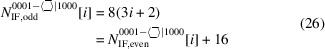




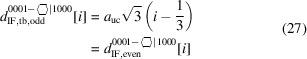




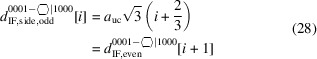




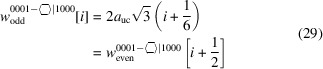




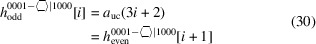




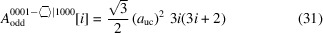

**Figure 6:**
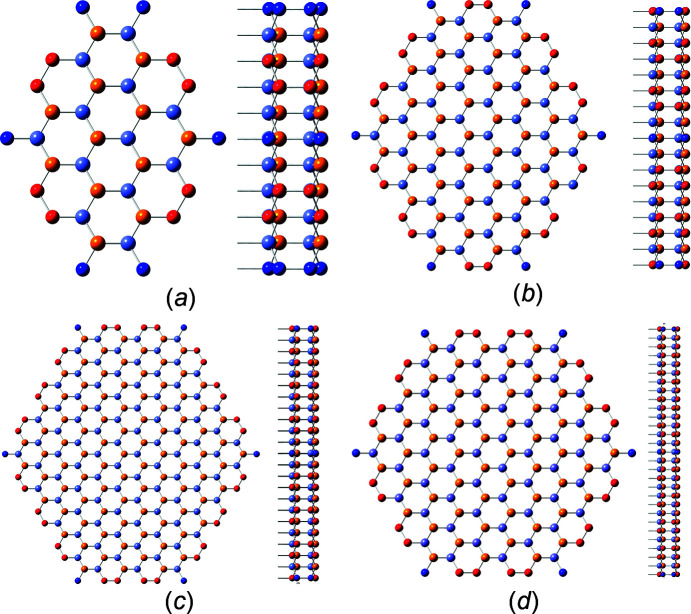
Cross section and side view of w-structured NWires with a [0001] growth axis, hexagonal cross section and {1000}-oriented interfaces, *odd* series: (*a*) X_76_ (*i* = 1), (*b*) X_220_ (*i* = 2), (*c*) X_436_ (*i* = 3) and (*d*) X_724_ (*i* = 4). For atom colours, see Fig. 5 ▸.

The first four members of the *odd* number series are shown in Fig. 6 ▸.

### NWires growing along the 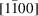 direction with a hexagonal cross section and four {0021} plus two {0001} interfaces

3.3.

**Figure 7:**
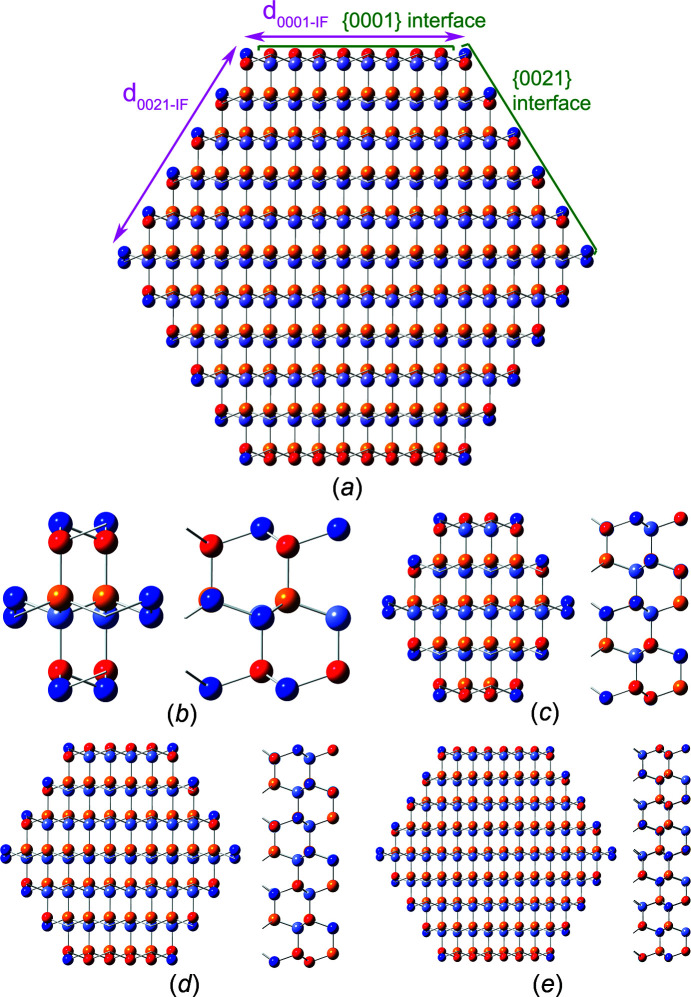
(*a*) Definition of characteristic lengths for the w-structured NWires growing along the 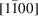 axis with a hexagonal cross section and two {1000} interfaces at the top and bottom, plus four side interfaces with a {0021} orientation, shown along with the assignment of the interface atoms to the respective interface plane. Top and side views of the first four members, *even* series: (*b*) X_16_ (*i* = 1), (*c*) X_56_ (*i* = 2), (*d*) X_120_ (*i* = 3) and (*e*) X_208_ (*i* = 4). For atom colours, see Fig. 5 ▸.

This cross section reveals the congruence between zb- and w-structures when seen along specific lattice vectors: the in-plane atomic arrangement, as seen along the 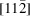 growth vector for the zb-structure, and along the 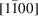 growth vector for w-structures, become indistinguishable. We encourage the reader to compare the top views of the NWire cross sections in Figs. 7 ▸ and 8 ▸ with those in Figs. 6 ▸ and 7 ▸ of König & Smith (2019 ▸, 2021 ▸). From the side view of the cross sections in Figs. 7 ▸ and 8 ▸, and the mentioned figures in König & Smith (2019 ▸, 2021 ▸), it becomes apparent that the sequence of atomic planes is *ABABAB* for the w-structure, while it is *ABCABC* for the zb-structure. Since these atomic planes run *orthogonal* to the cross section plane, such differences in the sequencing of atomic planes have no effect, resulting in identical projections of the w- and zb-structures with 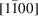 and 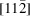 growth vectors, respectively. As a result, all number series for this w-NWire cross section are identical to the zb-NWire cross section with a 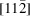 growth vector and two {111} plus four 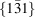 interfaces in König & Smith (2019 ▸, 2021 ▸), apart from structure-specific gauge factors for characteristic lengths and the area of the cross section.

As was the case in Section 3.2, no offsets in characteristic lengths or cross section area exist. Derivations for increments in specific lengths and cross section area can be found in Appendix *B* [App appb]. Despite the tangled lattice vectors for growth and interface orientations, such derivations are more straightforward as com­pared to both cross sections for w-NWires with a [0001] growth vector. We start again with the *even* series of this cross section. 

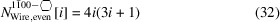




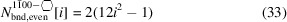




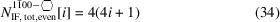




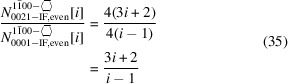

The first line in Equation 35 shows the explicit results per interface orientation, while the second line provides the simplified ratio. 

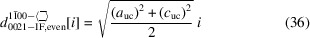




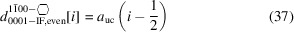




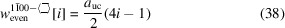




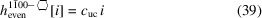




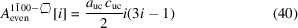

The definition of interface boundaries for the calculation of characteristic lengths and the cross section area, and the assignment of interface atoms to the respective interface plane are shown in Fig. 7 ▸, together with top and side views of cross sections for the first four members of the *even* number series.

We now list the *odd* series of the cross sections with a 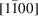 growth vector and four {0021} plus two {0001} interfaces. 

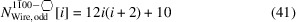




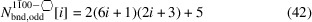




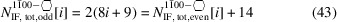




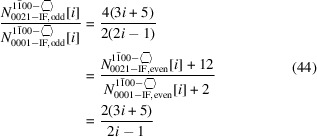

The first line in Equation 44 shows the explicit results per interface orientation, while the last line provides the simplified ratio. 

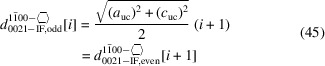




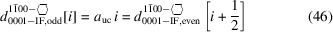




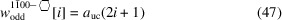




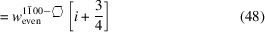




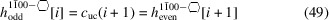




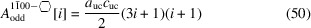

**Figure 8:**
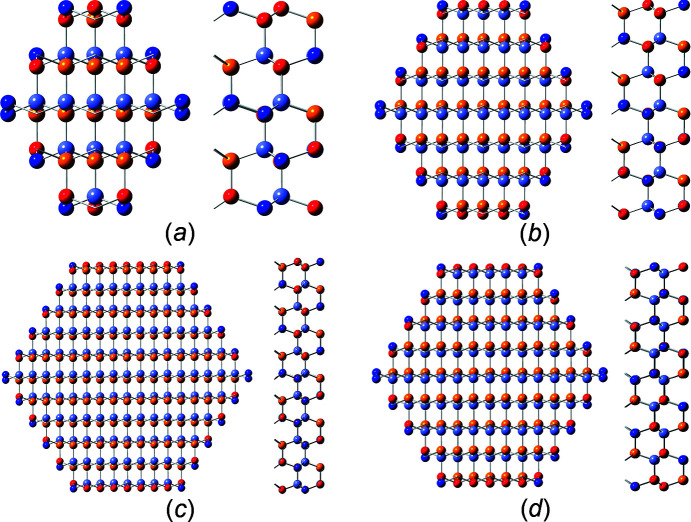
Cross section and side view of w-structured NWires growing along the 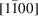 axis with a hexagonal cross section and two {0001} interfaces at the top and bottom, plus four side interfaces with a {0021} orientation, *odd* series: (*a*) X_46_ (*i* = 1), (*b*) X_106_ (*i* = 2), (*c*) X_190_ (*i* = 3) and (*d*) X_298_ (*i* = 4). For atom colours, see Fig. 5 ▸.

The first four members of the *odd* number series are shown in Fig. 8 ▸.

## 4. Usage of number series ratios on nanowire cross sections

The primary parameters of interest are the number of atoms within the NWire cross section, 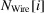 , the number of bonds between such atoms, 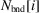 , and the total number of interface bonds, 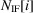 . The width, height and interface lengths of NWire cross sections serve mostly as a metric pointer to pick the right run index *i* for arriving at the correct description of the above variables in accord with experimental data (images with atomic resolution). The cross section areas allow the calculation of the areal densities of the electric or thermal currents, which allows for a direct comparison between different NWire sizes and crystallographic orientations. From the three primary parameters we listed above, we can form the ratios 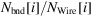 , 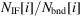 and 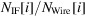 , all of which can be compared to each other by their respective 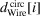 . Several research groups (Shtrikman *et al.*, 2009 ▸; Zardo *et al.*, 2009 ▸; Dubrovskii & Sibirev, 2008 ▸) obtained NWire diameters in the range 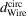 = 20–40 nm as an upper size limit for the wurtzite structure, below which it is (meta-)stable, converting to the zincblende structure for bigger diameters. Therefore, we limit the plotting of 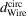 to a maximum of 40 nm which allows the data for ultrasmall diameters to be assessed in more detail.

**Figure 9:**
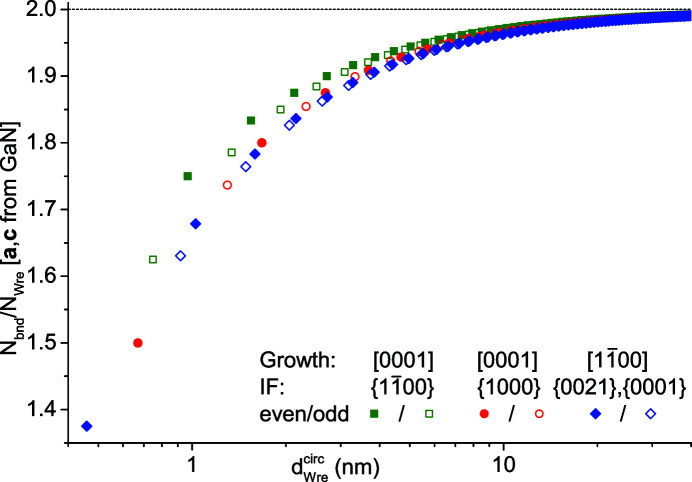
Ratio of NWire-internal bonds to NWire atoms 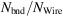 shown for all three NWire cross sections as a function of the NWire diameter 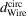 . We chose the lattice parameters of GaN; see text for details.

We start with the ratio 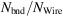 , which describes the number of NWire-internal bonds per NWire atom. This ratio converges to 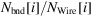 → 2 for *i* → ∞, as becomes evident from Fig. 1 ▸; each atom has four bonds, whereby each bond is shared with a first next-neighbour (1-nn) atom; 4/2 = 2 if the w-structure is infinite (and thus no bonds are ‘lost’ to any interfaces). The ratio 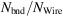 is a good gauge of the internal stress of an NWire, *e.g.* to counteract external forces from a substrate or shell, or for the resistance to integrate foreign atoms such as dopants onto lattice sites. In an inverse manner, 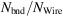 can serve as a precise guide for predicting stress propagation and a transfer of the crystallographic structure onto NWire shells as a consequence, such as for w-Si grown around zb-InP NWires (Algra *et al.*, 2011 ▸). The ratio 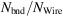 as a function of 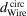 is shown for w-GaN NWires as an example in Fig. 9 ▸, whereby we used the unit-cell parameters *a* = 3.1891 Å and *c* = 5.1855 Å (Adachi, 2004 ▸).

As 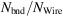 decreases for shrinking diameters 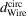 , the ability of the NWire to counterbalance external stress – or to exert crystallographic information on a shell material – increases. This statement originates from the number of bonds per NWire atom 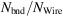 which can tolerate stress. Lower values of 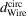 decrease 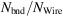 , thus increasing the stress per NWire-internal bond. Thereby, a build-up of counter-stress occurs until a certain stress limit of the NWire is exceeded, leading to structural defects, such as stacking faults and grain boundaries, eventually rendering the NWire polycrystalline. Experimental evidence for the above argument exists on a general basis for Si-NWires and Si nanocrystals, where the incorporation of foreign atoms onto lattice sites becomes increasingly unlikely for shrinking 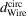 (Stegner *et al.*, 2009 ▸; Björk *et al.*, 2009 ▸), with a hard limit of 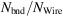 = 1.94 ± 0.01 for both NWires and nanocrystals (König & Smith, 2021 ▸). This process is called self-purification (Dalpian & Chelikowsky, 2006 ▸, 2008 ▸).

From Fig. 9 ▸ we see that NWires with a [0001] growth vector and 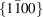 interfaces behave differently. The values of 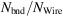 are significantly lower when compared to the other two w-NWire types, which have very similar values of 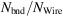 over 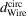 . From that observation, we can establish two hypotheses when considering NWires with similar 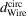 values. One, NWires with a [0001] growth vector and 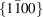 interfaces should be more vulnerable to external stress, or – in reverse – are less likely to imprint their crystallographic information onto an epitaxial shell material. Two, any incorporation of foreign atoms onto lattice sites in NWires with a [0001] growth vector and 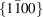 interfaces will be more likely compared to the other two NWire types. From a higher ratio of 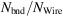 , we can also deduce that we obtain a smaller minimum 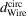 below which the NWires with a [0001] growth vector and 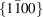 interfaces would suffer from significant structural defect densities and eventually significant amorphization. As for structural arguments, NWires with a [0001] growth vector and 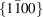 interfaces should be the most stable NWire type.

**Figure 10:**
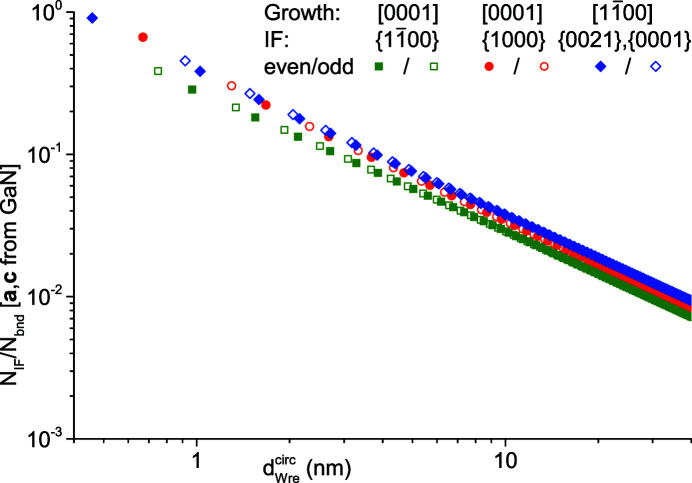
Ratio of interface bonds to NWire-internal bonds 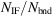 shown for all three NWire cross sections as a function of the NWire diameter 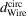 . We chose the unit-cell parameters of GaN; see text for details.

The ratio of interface bonds to NWire-internal bonds 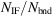 is a structural parameter similar to the ratio 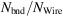 , though here the key information is the inclusion of the interface as the coupling means between the NWire and its environment. Therefore, 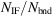 presents a gauge for the *static and dynamic stress transfer over the interface*. Naturally, 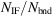 declines for increasing 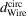 , eventually converging to 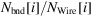 → 0 for *i* → ∞. We can obtain the respective gradient by which 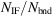 decreases for sufficiently large *i* from the respective equations for 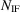 and 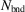 , namely, their leading terms in powers of *i*. Ordered by gradient, we get 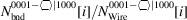 = 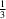 *i* ^−1^, 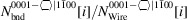 = 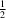 *i* ^−1^ and 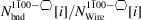 = 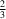 *i* ^−1^. As becomes apparent from Fig. 10 ▸, such gradients do not appear to play a major role for 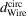 ≤ 40 nm. For metastable crystallographic systems such as NWires, a threshold for 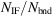 exists below which structural defects start to occur at or in the vicinity of interfaces which represent the weakest link in the crystallographic construct. From Fig. 10 ▸, we see that 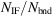 of the cross section with a [0001] growth vector and 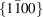 interfaces has lower values as compared to the two other NWire types. One origin of this finding follows straight from the higher number of internal bonds per NWire atom 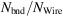 , leaving less bonds available to the interface. Another contribution arises from the lower number of interface bonds per 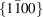 interface 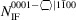 , followed by the value of {1000} interfaces 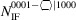 , and eventually by the {0021}-dominated interface bond densities 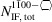 . With the lowest 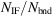 values for NWires with a [0001] growth vector and 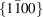 interfaces, such NWires are more likely to possess interface defects: more internal bonds exist per interface bond to counteract stress between the NWire and its environment. A few minor features exist in Fig. 10 ▸. From Equations 4, 11, 19 and 26, it follows that 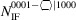 → 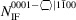 for *i* and consequently 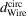 → ∞, since 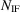 differs only by a constant given by the 12 corner atoms with two interface bonds each of the cross section with a [0001] growth vector and {1000} interfaces. The values of 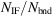 are furthermore important for phonon propagation and reflection, a feature important for nanoscopic thermal transport relevant for heat dissipation (Vázquez *et al.*, 2009 ▸), thermoelectrics (Dubi & Ventra, 2011 ▸) or hot carrier photovoltaics (König *et al.*, 2020 ▸).

**Figure 11:**
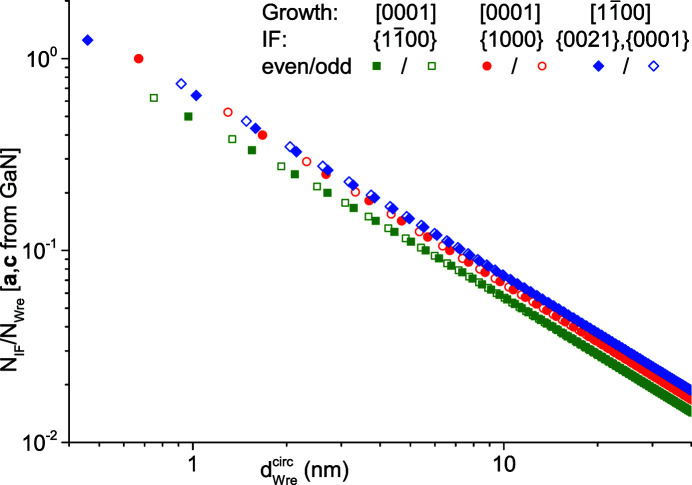
Ratio of interface bonds to NWire atoms 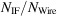 shown for all three NWire cross sections as a function of the NWire diameter 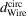 . We chose the unit-cell parameters of GaN; see text for details.

The last ratio we look at is the number of interface bonds per NWire atom, 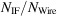 . This ratio describes the number of electronic ‘delivery channels’ per NWire atom, and thus the *structural* ability (*versus* quantum-chemical ability) of the NWire to acquire or deliver electronic charge from or to its environment, respectively, by charge transfer. Such transfers occur *via* interface dipoles (Campbell *et al.*, 1996 ▸), the pillow effect (Otero *et al.*, 2017 ▸) or the NESSIAS effect (König *et al.*, 2021 ▸). Fig. 11 ▸ shows the values of all cross sections as a function of 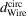 .

The cross section with a [1000] growth vector and 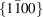 interfaces yields the lowest values of 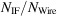 per 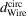 . The values for the remaining two cross sections are virtually identical for ultrathin NWires with 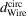 ≤ 3 nm. Then, 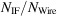 of the cross section with a 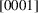 growth vector and {1000} interfaces gets smaller, reaching *ca*. 90% of the value obtained for the cross section with a 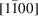 growth vector and two {0001} plus four {0021} interfaces for 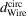 = 30–40 nm. This finding indicates that the interface presents less of a bottleneck to charge transfer for the latter NWire class. We can thus expect a charge transfer which affects NWire atoms being located further towards the centre of the cross section of such NWires, and consequently a larger NWire diameter up to which the NESSIAS effect occurs at full scale.

## 5. Conclusions

We have deduced analytical number series for w-structured NWires as a function of diameter and interface faceting, featuring regular hexagonal cross sections with a [0001] growth vector and six 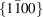 interfaces, regular hexagonal cross sections with a [0001] growth vector and six {1000} interfaces, and nonregular hexagonal cross sections with a 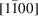 growth vector and two {0001} plus four {0021} interfaces. All cross sections are presented in an *even* and an *odd* scheme to facilitate matching to different symmetry centres encountered experimentally. The calculated parameters are the number of NWire atoms 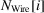 , the number of bonds between such atoms 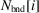 and the number of NWire interface bonds 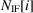 , the interface lengths 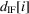 , the cross section widths 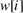 , the heights 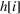 and the total cross section areas 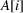 . All expressions are linked to NWire spherical diameters 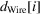 to enable a direct parameter comparison between different morphologies.

Geometrical details of the derivation of increments and offsets for area and interface lengths, as well as heights and width, of all cross sections are provided in the Appendix sections to facilitate a retracing of the number series, com­plemented by a complete derivation of all *even* number series for cross sections with a [0001] growth vector and six 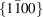 interfaces.

From the three atomistic parameters 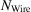 , 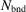 and 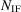 , three ratios were shown to yield valuable structural information for w-NWires, extending to electronic applications. The ratio 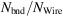 is useful to gauge the internal stress of NWires, which is key in the evaluation of self-purification and dopant segregation as encountered in impurity doping, and the general stress response of NWires to an external force. Both 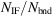 and 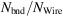 can be applied to optical spectroscopy methods, such as FT–IR, Raman, photoluminescence or electroluminescence, to interpret and deconvolute spectra into NWire-immanent (internal) and matrix/shell (external) components. The ratio 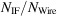 describes the electronic interaction of NWires with the embedding matrix or ligands to gauge the impact of interface dipoles or interface charge transfer on the NC electronic structure.

As noted for our work on zb- and diamond-structured NWire cross sections, the analytic description of w-NWire cross sections provides a major advance in experimental data interpretation and the understanding of III–V, II–VI and group IV-based w-NWires. In more detail, the number series allows for a deconvolution of the experimental data into environment-exerted, interface-related and NC-internal phenomena. The predictive power of our method could render it an essential tool in the prediction of NWire cross sections and in tuning the processing conditions for tailoring NWires towards desired shapes and interface properties.

We plan to publish a fourth article shortly which will introduce cross-section morphing into arbitrary convex shapes of the w-NWire cross sections introduced herein, again in analogy to our works on zb-/diamond-structure NWires (König & Smith, 2022 ▸). To this end, experimental data can be interpreted with high accuracy as, to the best of our knowledge with respect to the current state of the art, no data on w-structure NWires with regular cross sections have been published.

## Figures and Tables

**Figure 12:**
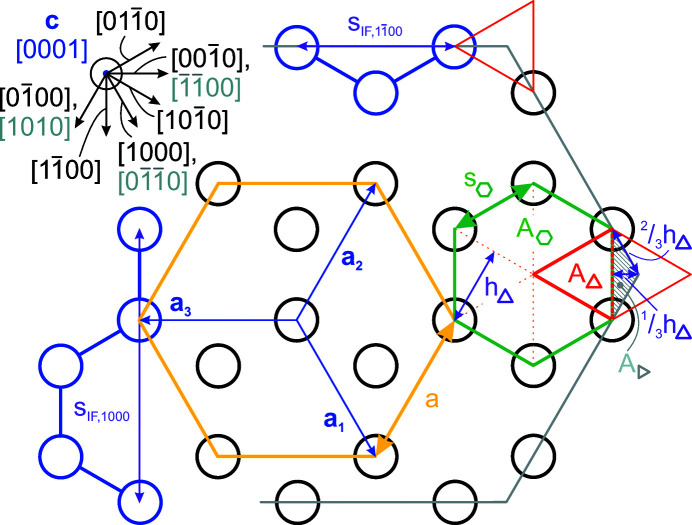
Geometric relations for the wurtzite lattice structure along the **c** = [0001] growth vector. The unit cell in the {0001} plane is shown in yellow and its relevant lattice vectors **a**_1_, **a**_2_ and **a**_3_ are shown in blue. The unit area 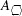 (grey–green) for the {0001} plane is defined by the area of a six-membered ring, consisting of six equilateral triangles 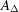 = 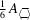 (red). The distance increments required for calculating the lengths of the {1000} and 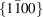 interfaces are shown by 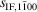 and 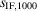 , respectively, with local atomic bonds shown. As an auxiliary parameter, we show the height 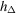 and its relevant fractions of the congruent equilateral triangles. All length parameters other than the lattice vectors are shown in purple. Grey lines show the 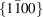 interfaces, which require some additional derivations in terms of fractional 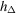 and a fractional area 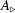 = 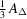 . A scheme of relevant lattice vectors within the {0001} plane is shown on the upper left, with **c** being orthogonal to the {0001} plane; indices shown in grey present alternative combinations of lattice vectors.

**Figure 13:**
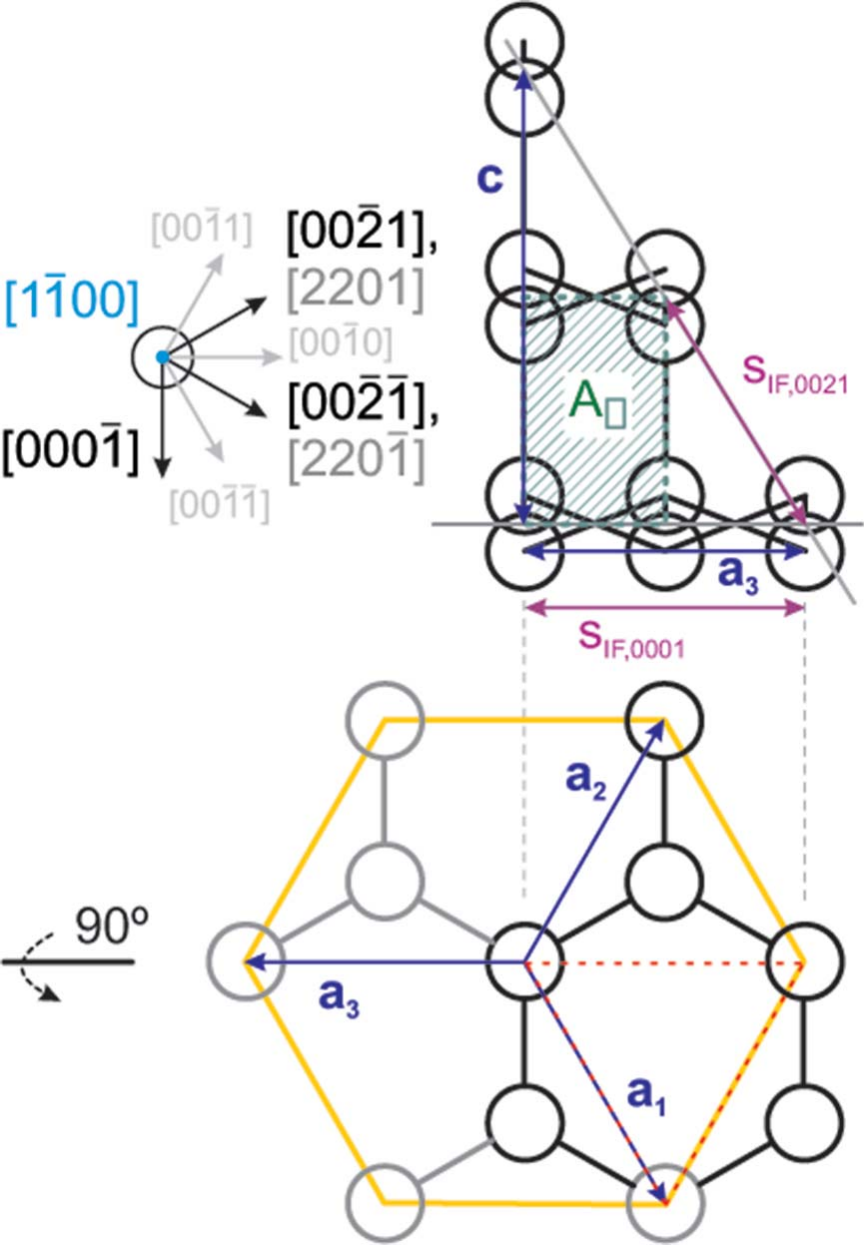
Geometric relations for the wurtzite lattice structure along the 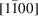 growth vector. The lower graph shows the same lattice arrangement turned 90° clockwise around the horizonal axis of 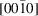 orientation, with grey atoms and bonds added to facilitate comparison with Fig. 12 ▸. Lattice vectors are shown in blue, whereby only **a**_3_ and **c** are relevant in the 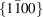 plane. For {0021} interfaces, **a**_3_ can be alternatively described by a combination of **a**_1_ and **a**_2_, again shown in grey underneath the orthogonal vector of the respective interface on the left side of the top graph. Orthogonal vectors which are not part of the {0021} interface class are shown by grey arrows and respective indices in smaller grey print. Distance increments for the {0021} interfaces *s*_IF,0021_ and the {0001} interfaces *s*_IF,0001_ are shown in purple. The unit area for this cross section *A* 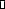 is shown as a hatched grey–green rectangle.

**Table 1:** Slab thickness 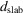 of NWire cross sections as a function of growth-axis orientation given in unit-cell (UC) lengths per growth orientation to achieve periodicity; numbers of atoms and of bonds per column as described per feature seen in cross section top view are given to enable the counting of atoms and NWire-internal bonds

Growth axis	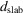	Atoms per column in top view	Bonds in top view
0001	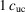	2	1 per column, 2 per —, 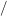 and 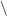
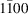	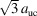	1	2 per 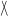 , 1 per — and –†

† Bond symbols must be turned by 90° to align with the graphs in Figs. 6 ▸, 7 ▸ and 13 ▸

**Table 2:** The parameter list for each NWire cross section; all parameters are calculated per NWire slab

Parameter	Description
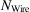	No. of atoms forming NWire
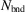	No. of bonds within NWire
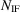	No. of interface (IF) bonds of NWire
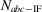	No. of bonds per IF type 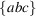
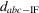	Length of IF with orientation 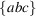
*w*	Maximum width of NWire cross section
*h*	Maximum height of NWire cross section
*A*	Cross section area

**Table 3:** List of NWire shape indices [cross section, growth direction and side interfaces (where necessary)] added to all parameters as a superscript

Superscript	Growth axis	Cross section shape	Side interfaces†
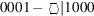	0001	Hexagon	1000
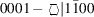	0001	Hexagon	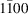
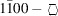	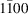	Hexagon	

† Only when required to distinguish cross sections.

**Table 4:** 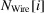
 presented by atoms per atom row of the respective NWire cross section per run index *i*. The second column shows the atoms per atom row of the respective NWire cross section per run index *i* – *cf.* Figs. 3 ▸(*b*) to 3(*d*) for *i* = 1 to 4, and Fig. 3 ▸(*a*) for *i* = 6. The third column contains the sum of all atoms per NWire cross section 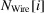 , the fourth column its first-order difference quotient and the fifth column its second-order difference quotient

*i*	Atoms per row	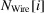	d 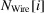 /d*i*	d 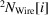 /d 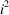
1	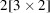	12		
			36	
2	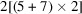	48		24
			60	
3	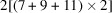	108		24
			84	
4	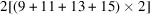	192		24
			108	
5	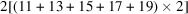	300		24
			132	
6	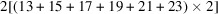	432		…
			…	
…	…	…		

**Table 5:** 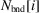
 presented by bonds per atom row of the respective NWire cross section per run index *i*. The second column shows the bonds per atom row of the respective NWire cross section per run index *i* – *cf.* Figs. 3 ▸(*b*) to 3(*d*) for *i* = 1 to 4, and to Fig. 3 ▸(*a*) for *i* = 6. The first summand refers to one bond per atom column and is thus = 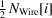 , *cf.* Table 1 ▸. The last summand at the closing square bracket accounts for half of the bonds in the centre of the respective cross section. These bonds are multiplied by two, as are the bonds in the term (…) × 2, the latter presenting the total number of bonds between atom columns of one half of the cross section apart from its centre. The third column contains the sum of all internal bonds per NWire 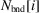 , the fourth column its first-order difference quotient and the fifth column its second-order difference quotient

*i*	Bonds per atom row	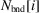	d 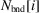 /d*i*	d 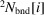 /d 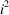
1	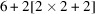	18		
			66	
2	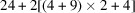	84		48
			114	
3	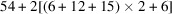	198		48
			162	
4	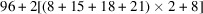	360		48
			210	
5	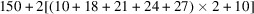	570		48
			258	
6	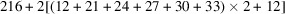	828		…
			…	
…	…	…		

**Table 6:** 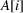
 and its difference quotients presented in units of 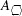 . See Appendix *A* [App appa] for the definition of 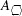 , and Figs. 3 ▸(*b*) to 3(*d*) for *i* = 1 to 4, and Fig. 3 ▸(*a*) for *i* = 6. The second column shows the detailed scheme and its components are (from left to right): 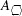 per row of hexagonal areas for one half of the cross section, apart from the centre row, multiplied by two (which includes the other half up to the centre row), 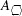 of the centre row, 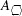 of the isoceles triangles at the six interfaces and 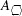 of the offset area due to the six small isoceles triangles at the six corners; see Fig. 12 ▸ for details. The third column contains the sum of all unit areas 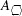 of 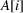 , the fourth column its first-order difference quotient and the fifth column its second-order difference quotient

*i*	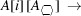	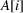	d 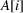 /d*i*	d 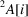 /d 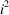
1	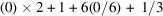	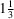		
			7	
2	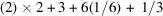	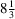		6
			13	
3	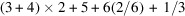	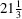		6
			19	
4	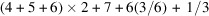	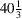		6
			25	
5	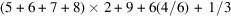	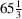		6
			31	
6	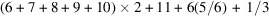	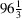		…
			…	
…	…	…		

## Appendix A Geometric details for NWire cross sections with a [0001] growth vector

The cross sections of NWires growing along the **c** = [0001] vector naturally spare **c** from the geometrical analysis which proceeds *via* the in-plane lattice vectors of the cross section **a**_1_, **a**_2_ and **a**_3_ (see Fig. 2 ▸).

We start the analysis with the w-UC projection into the {0001} plane defining the NWire cross sections (see Fig. 12 ▸), introducing the lattice constant  in the process. From Fig. 12 ▸ we see immediately that the side length of this UC projection is also the increment  in  interface length. The unit area we use for describing the cross section area  is represented by a six-membered Ga_3_N_3_ ring, shown in grey–green in Fig. 12 ▸. Both hexagons are regular and thus similar, thereby simplifying the analysis considerably. Regular hexagons are composed of six equilateral triangles with the area  , which is another property we put to good use. From the atomic arrangement of the w-structure in the {0001} plane, we see that the distance of two parallel sides of six-membered ring describing  is identical to the side length of the regular hexagon given by the w-UC projection onto the {0001} plane. This relationship translates into twice the height of the equilateral triangles. The height of an equilateral triangle is given from Pythagoras’ theorem as  , with  being the side length of the equilateral triangle with the area  , as well as the side length of the six-membered hexagonal ring. From the above considerations, we see immediately that  = *a*/2, and hence  . With  as the side length and  as the height of the equilateral triangle, we get its area  . From this result, it follows instantly that the unit area is  . For cross sections with  interfaces, there are six offset areas  occurring at every corner; see Figs. 3 ▸ and 12 ▸. Its calculation is once again straightforward by virtue of the hexagonal symmetry, rendering any of three symmetry axes of the equilateral triangles parallel to the vector class of  and thus  . It follows from straightforward symmetry arguments of equilateral triangles that the area of any of the three isoceles triangles emerging by said areal decomposition is  . Since we have six corners with an offset area  each, the total offset area for NWire cross sections with  interfaces is  . The offset length of the  interfaces becomes apparent when looking at the isoceles triangle manifesting  , amounting to  . The offset length for the width of cross sections with  interfaces follows straight from symmetry arguments (see Fig. 12 ▸) as  =  .

We begin the derivation of relevant characteristic lengths for the cross section with {1000} interfaces with the increment of {1000} interface length  , which corresponds to the distance of two parallel sides of the w-UC projected onto the {0001} plane. This length is equivalent to twice the height of the six equilateral triangles composing the w-UC projected onto the {0001} plane, hence  . With no offset areas or lengths existing for cross sections with {1000} interfaces, our geometrical analysis for NWire cross sections with a [0001] growth vector is complete.

## Appendix B Geometric details for NWire cross sections with a growth vector and two {0001} plus four {0021} interfaces

Counterintuitively, the geometric details of the cross section for NWires growing along the [  ] vector with  interfaces is simpler yet compared to the geometric derivations above. Fig. 13 ▸ shows a small portion of the cross section in the top graph, complemented by the lattice structure turned by 90° to expose the connection to the symmetry considerations carried out above for the NWire cross sections with a [0001] growth vector. The layout of this NWire cross section is defined by two lattice vectors, namely,  and **c** lying in the  plane. An alternative description for **a**_3_ is given by **a**_3_ = −**a**_1_ − **a**_2_, *cf.* Fig. 2 ▸; such indices are shown in grey in Fig. 13 ▸. Comparing the cross sections shown in Fig. 7 ▸ with Fig. 13 ▸, we see immediately that the increment of the {0001} interface length is equal to the increment of the {1000} interface length, *viz.*  =  = *a*. From a comparison of Fig. 7 ▸ with Fig. 13 ▸, we see that the increment in width is equal to  . The increment for the {0021} interface lengths follows in a straightforward manner from Pythagoras’ theorem and half the length of each in-plane lattice vector as  , describing the diagonal of a rectangular unit area which in turn defines the unit area for this cross section, namely, *A*  =  . Finally, the increment in height is given by the value of the [0001] lattice vector *c*, describing twice the distance between two adjacent corrugated atomic planes in the **c** direction.

## Appendix C Derivation of the number series explained with respect to the NWire cross sections with a [0001] growth vector and six interfaces, *even* series

We start with the variables which show a quadratic dependence on the run index *i* and thus the NWire diameter  , listing their evolution with *i* in tabular form from which we derive their number series. Variables with linear dependence on *i*, such as specific lengths, are straightforward to derive and a description is provided at the end of this section. We start with the number series describing the amount of NWire-internal atoms  .

From Table 4 ▸ we see that d  /d*i* increases by 24 for each *i* → *i* + 1, and that an offset of −12 exists for d  /d*i*, whereby we obtain

These findings can be easily verified by examining the fourth column in Table 4 ▸, where d  /d  , d  /d  , *etc*. In order to obtain an analytic description of  , we have to sum up all d  /d*i*, resulting in the Riemann sum (Zeidler *et al.*, 2004 ▸)

Equation 52 presents an integration over discrete points. As such, there may be an integration constant coming into existence as discussed below. The summation itself can be expressed in a sum formula as per the exponent of *i*:

In Equation 53, we made use of the relationship (Zeidler *et al.*, 2004 ▸)

The solution for *i* ^0^ is trivial, as the constant offset in d  /d*i* is just multiplied by *i* in accord with an integration of a constant; see Equation 53. When solving Equation 53, we obtain  = 12,  = 48,  = 108,  = 192,  = 300,  = 432, *etc*. We see that Equation 53 has a zero integration constant and thus already presents the final solution of  as per Equation 2.

Finding the number of NWire-internal bonds  is not quite as straightforward; we follow the same scheme as used for  . There is one bond and two atoms per atom column, *cf.* Table 1 ▸, yielding the first summand in the total sum of the internal NWire bonds as  . We then continue by listing the bonds per atom row, including the vertical bonds to the next smaller atom row once we have left the interface. The latter values are the small numbers at the left round bracket, *i.e.* 4 for *i* = 2, 6 for *i* = 3, 8 for *i* = 4, *etc*. Then we take the bonds of each atom row plus its bonds to the next smaller atom row until we reach the atom row at the centre. These numbers of internal bonds increase by an increment of three when moving towards the centre of the cross section and are listed within the round brackets per cross section in Table 5 ▸. Since we have two bonds between each atom column, we multiply the above values by 2, yielding the term (…) × 2. The bonds in the centre of the cross section are counted only once (so are not multiplied by 2 as all off-centre values) to arrive at exactly all bonds between atom columns for one half of the cross section, being described by the term […]. This term is then multiplied by two to cover the entire cross section, apart from the bonds per atom column being presented by the first summand as described above. Table 5 ▸ presents the scheme together with the total value of  , plus its first- and second-order difference quotient.

While we here follow the same scheme as for  , we choose a slightly different way of calculating  from Table 5 ▸ which does not rely as much on the intuitive discovery of d  /d*i*, albeit being less concise.

We note from Table 5 ▸ that d  /d  , from which it follows by discrete integration that d  /d  , with  being the integration constant as discussed above for  . For now, we ignore  and convert d  /d*i* directly *via* Equation 54 to

When calculating  with Equation 55, we see that there is a difference to the full solution in Table 5 ▸, accounting for the integration constant  . The difference of row three in Table 5 ▸ to Equation 55 is  = 18 − 48 = −30,  = 84 − 144 = −60,  = 198 − 288 = −90,  = 360 − 480 = −120, or generally  . This solution must be added to Equation 55 to arrive at the exact solution given by Equation 3, *viz.*

The third property with a quadratic dependence on *i* is the cross section area  . To this end, we describe  by the number of areas rendered by hexagonal rings of the w-lattice structure when seen along the [0001] growth vector, hence in units of  . All nonhexagonal areas, such as the bigger isoceles triangles at the interfaces and the six small isoceles triangles at the six corners which we count as an offset area (being constant  ), can be converted into units of  ; see Appendix *A* [App appa] for details. Table 6 ▸ shows the relevant development of  with its first- and second-order difference quotient.

We integrate d  /d  , yielding d  /d  . This result comes about by the first value of d  /d  occurring for *i* = 2. We therefore obtain a correct solution for *i* = 2 under the constraint of the integration term 6*i* if we introduce an integration constant of  = −5, as is evident from d  /d  , d  /d  , d  /d  , *etc*. Once again, we make use of Equation 54, obtaining

With  derived in Appendix *A* [App appa], we arrive at Equation 8 in Section 3.1.

The remaining variables –  ,  ,  and  – show a linear dependence on *i*. Due to the hexagonal symmetry, we consider only one interface and multiply its result by six. We see from Fig. 3 ▸ that all interface atoms have one interface bond. These atoms increase by two for each increment of *i*, namely, by one atom column which contains two atoms, *cf.* Table 1 ▸, arriving at  , as presented by Equation 4 in Section 3.1. For  , we use its increment  over *i* and its offset value  ; see Appendix *A* [App appa] for details. We see from Fig. 3 ▸ that the number of  required to describe  is always *i* − 1 plus  , resulting in  , and generally in *d*_IF_ =  = *a*(*i* −  ) (see Equation 5 and Section 3.1). The width of the cross section  is given by the distance between two of its opposite corners and has an offset of  (see Fig. 12 ▸ and Appendix *A* [App appa]). For *i* = 1, we have *w*[*i* = 1] =  . With every subsequent increment in *i*, we add  to  . We thus arrive at *w*[*i*] = *h*_  _(4*i* − 2) + *w*_offset_ =  , which can be further simplified into  , arriving at Equation 6 in Section 3.1. The height of the cross section, which is the distance between two parallel interfaces, starts with the distance between two opposite corners of the hexagonal ring, as is obvious from Fig. 3 ▸(*b*),  ; see also Appendix *A* [App appa] for details. With every subsequent increment in *i*, such a hexagonal ring plus one adjacent bond is added, amounting to  , which is equivalent to the increment of the {1000} interfaces  (see Fig. 12 ▸). We thus arrive at *h*[*i*] =  =  , as presented by Equation 7 in Section 3.1.
